# Acute pancreatitis in a young woman with COVID-19 infection: A case-report

**DOI:** 10.22088/cjim.12.0.474

**Published:** 2021

**Authors:** Masood Faghih Dinevari, Mehdi Rasoolimanesh, Mehdi Tarverdizadeh, Ali Riazi, Samaneh Abbasian, Aysan Zeinolabedini, Sina Hassannezhad

**Affiliations:** 1 Liver and Gastrointestinal Diseases Research Center, Tabriz University of Medical Sciences, Tabriz, Iran; 2 Student Research Committee, Tabriz University of Medical Sciences, Tabriz, Iran

**Keywords:** COVID-19, Acute pancreatitis, Case report

## Abstract

**Background:**

Little is known about the development of acute pancreatitis as a complication of corona virus disease of 2019 (COVID-19) infection. This case report describes the presentation of acute pancreatitis in a young woman who then was diagnosed with COVID-19 infection.

**Case Presentation:**

An 18-year old previously healthy woman referred to Imam Raza hospital, Tabriz, Iran with a 3-day history of intermittent and crampy abdominal pain. She had serum amylase of 1288 IU/L and serum lipase of 1541 IU/L. She was diagnosed with acute pancreatitis. She was instructed nil per os (NPO) and serum therapy and also was given pantoprazole, and pethidine for her pain management. The laboratory tests for assessing the etiology of acute pancreatitis were normal. Abdominal and pelvic spiral computed tomography (CT) scan revealed edematous pancreas and enhancing loculi fluid accumulation around pancreas along with the small amount of ascites fluid that all suggest acute pancreatitis. Due to the presentation of fever and COVID-19 pandemic and her potential society exposure, we tested SARS CoV-2 by polymerase chain reaction which was positive. The blood C-reactive protein (CRP) level was 3+ but the chest x-ray showed no findings compatible with COVID-19. Eventually after receiving conservative therapy for her pancreatitis, she was discharged from hospital in the good general condition and she has not experienced any episodes of abdominal pain again.

**Conclusion:**

This case highlights acute pancreatitis as a suspected complication associated with COVID-19 and the need for further research.

On Dec** 31, 2019,** Wuhan Municipal Health Commission, China, reported a cluster of cases of pneumonia in Wuhan, Hubei Province (1). A novel coronavirus was eventually identified, and soon the world has been challenged by severe acute respiratory syndrome coronavirus 2 (SARS CoV-2). Symptoms include fever (43.8%-88.7%) as the most common one, cough (67.8%), fatigue (38.1%), myalgia or arthralgia (14.9%), and shortness of breath (18.7%), (2, 3). The pathogenesis mechanism of coronavirus was attributed to attaching virus to angiotensin-converting enzyme 2 (ACE2) receptors of cells and via that it could enter host cells and begin to damage various tissues and organs. These receptors located at the lungs, heart, kidney, artery, and intestine (4). Previous studies reported some gastrointestinal-related symptoms of coronavirus such as diarrhea, anorexia, abdominal pain, nausea, and vomiting (5). Another site that postulated affected by coronavirus is the pancreas. 

Some viruses such as cytomegalovirus (CMV), human immunodeficiency virus (HIV), herpes simplex virus (HSV), Coxsackie virus, hepatotropic virus, mumps, and varicella-zoster virus were shown to cause infectious pancreatitis (6) (7) but little is known about SARS CoV-2 effects on the pancreas. So, in the present study, we reported the case of a woman complaining of abdominal pain who was diagnosed with acute pancreatitis. However, all the possible causes of pancreatitis were excluded. Due to the current COVID-19 pandemic and her fever and potential society exposure, we screened her for COVID-19 infection by polymerase chain reaction which was positive. This brings our suspicion about SARS CoV-2 as a potential cause of acute pancreatitis.

## Case presentation

An 18-year old previously healthy woman was admitted to the emergency department at Imam Reza Hospital affiliated to the Tabriz University of Medical Sciences, Iran, with a 3-day history of intermittent and crampy abdominal pain. It was aggravated during meals and diminished when the patient leaned forward and also radiated to the back. She also complained of nausea and vomiting. She was instructed nil per os (NPO) and serum therapy and was also given pantoprazole and pethidine for her pain management.

The patient was awake and hemodynamically stable and had a low-grade fever (37.6 Celsius axillary). There was no history of alcohol abuse, diabetes, biliary or peptic ulcer disease or trauma and the family history was unremarkable. She was taking no medications. Physical examination revealed a well-developed female in moderate distress. No sinus tenderness or nasal polyposis was noted. There was no submandibular and axillary lymphadenopathy. The lungs were clear. There was no digital clubbing. The cardiac examination was unremarkable. The abdominal examination revealed moderate epigastric tenderness. 

**Figure 1:**
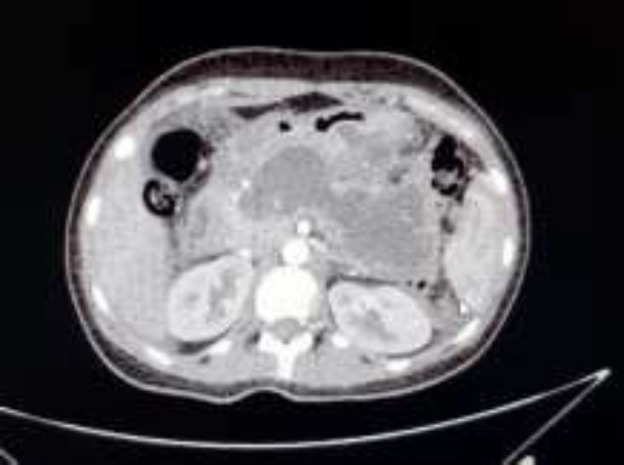
Abdominal CT scan: revealed edematous pancreas and enhancing loculi fluid accumulation around pancreas along with small amount of ascites fluid that all suggest acute pancreatitis

Serum biochemical analysis showed that her serum amylase was 1288 IU/L (normal up to 130 IU/L) and serum lipase was 1541 IU/L (normal up to 160 IU/L). So she was diagnosed with acute pancreatitis. The laboratory tests for assessing the etiology of acute pancreatitis including serum calcium, serum lipids, liver function tests, bilirubin and glucose, immunology tests, β-subunit of human chorionic gonadotropin (beta HCG), sweat test (for excluding cystic fibrosis as a rare cause of presentation), and complete blood count were obtained and were all normal. The electrolytes and blood cultures were also normal. The serum IgG4 level was obtained to assess autoimmune chronic pancreatitis (AIP), which was 1378 mg/L (normal 110-1570 mg/L) (Table 1). Abdominal and pelvic spiral CT scan (figure 1) revealed the edematous pancreas and enhancing loculi fluid accumulation around pancreas along with the small amount of ascites fluid that all suggest acute pancreatitis. 

**Figure 2:**
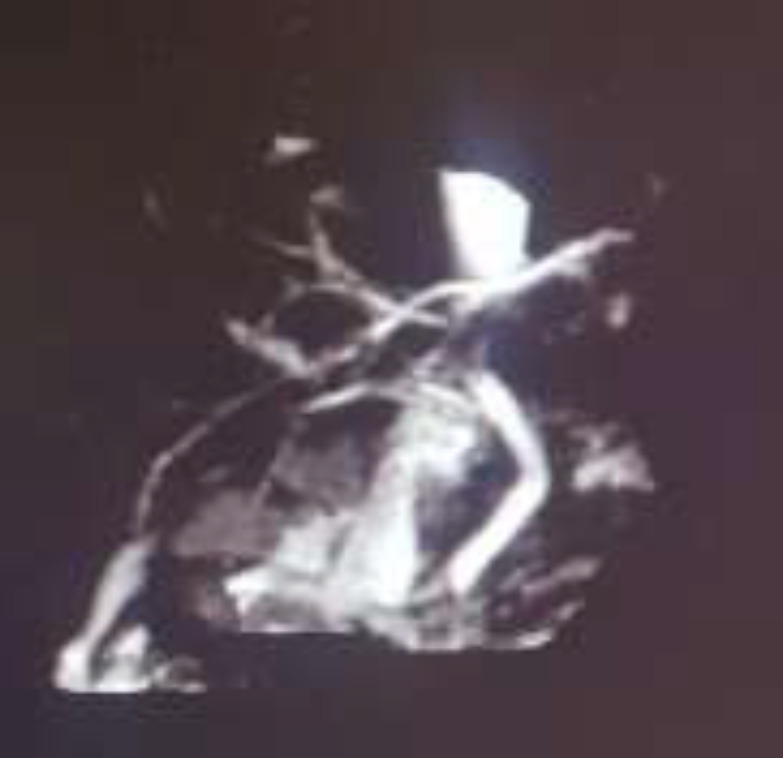
MRCP revealed the intrahepatic and extrahepatic bile ducts that showed normal caliber

We also obtained abdominal sonography that revealed no gallbladder stone but showed an edematous pancreas with anterior-posterior (AP) diameter of 4 cm which is suggestive of pancreatitis. Magnetic resonance cholangio pancreatography (MRCP) was performed at 1.5 Tesla scanner using the body coil that revealed the intrahepatic and extrahepatic bile ducts that showed normal caliber (figure 2). The gallbladder was unremarkable. 

**Figure 3:**
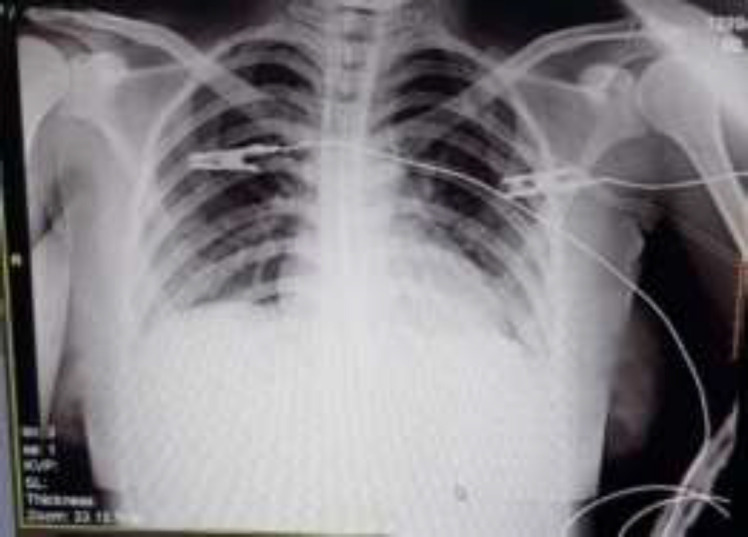
Chest x-ray no findings suggestive of COVID-19 pneumonia

Due to the presentation of fever and COVID-19 pandemic and her potential society exposure, the CRP (C-reactive protein) level was checked which was 3+. Moreover, the polymerase chain reaction (PCR) was performed in specimens taken from combined NPSs and throat swabs that were positive for SARS‐CoV‐2. Then we obtained chest x-ray which showed no findings compatible with COVID-19 (figure 3). 

**Figure 4:**
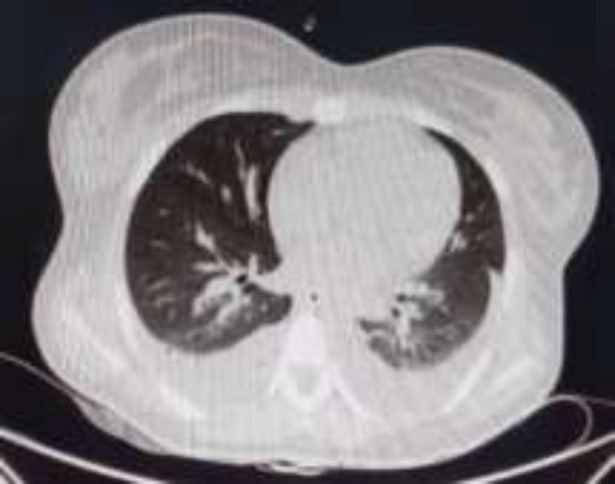
Chest cuts of abdominal CT scan revealed bilateral pleural effusion

**Figure 5:**
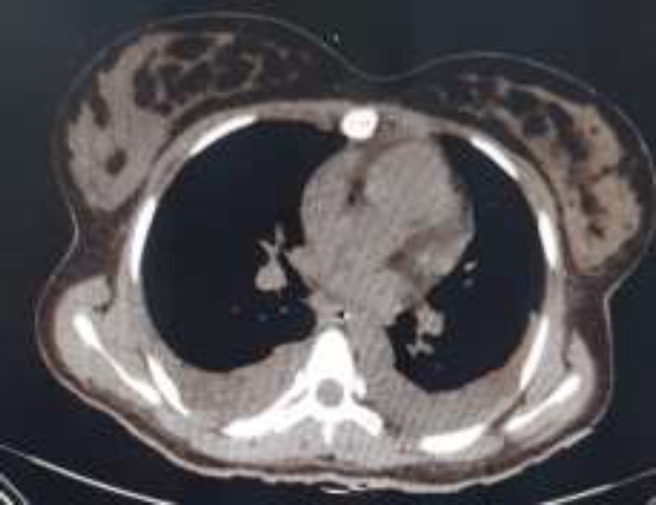
Chest cuts of abdominal CT scan revealed no findings suggestive for covid-19 pneumonia

In the implicit review of chest cuts of abdominal CT scan, mild bilateral pleural effusion was noticed but there was no evidence of covid-19 pneumonia (figures 4, 5). Eventually after receiving conservative therapy for her pancreatitis, she was discharged from hospital in good general condition and she has not experienced any episodes of abdominal pain again.

**Table 1:** Clinical Laboratory Result

	**Reference range**	**Day 1**	**Day 2**	**Day 3**	**Day 4**	**DAY 5**	**Day 6**
Hematocrit (%)	36-46	38.8	32.3	31.4			34.5
Hemoglobin(g/dl)	12_16	12.2	9.9	9.6			10.4
White cell count( per µL)	4000-11000	18800	8100	7100			4000
Segments (%)		88.2	75.6	71			57.1
Lymphocyte (%)		7.3	15.8	20.4			36.3
Mixed (%)		4.5	8.6	8.6			6.6
Platelet count (per µL)	150000-450000	576000		346000			381000
INR (Index)	0.9-1	1	1.34				
PT (Sec.)	Up to 13.4	12.5	18				
PTT (Sec.)	25-45	25	38				
Urea (mg/dl)	15-40	21	15		11		10
Creatinine (mg/dl)	0.7-1.4	0.8	0.7		0.61		0.73
Serum Sodium (mmol/L)	136-145	139	138		140		142
Serum Potassium (mmol/L)	3.6-5	4.6	4.2		4		4.1
Serum Magnesium(mmol/L)	1.8-2.6		1.9				
Blood sugar (mg/dl)	Up to 200	107			89		
Albumin (g/dl)	3.5-5.2		3.2				
Amylase (IU/L)	Up to 100	1288				244	
Lipase (IU/L)	Up to 60	1541				323	
SGOT (U/L)	0-37	29	19			29	
SGPT (IU/L)	0-31	28	24			21	
Total Bilirubin (mg/dl)	0.1-1.2	0.8	0.8				
Direct Bilirubin (mg/dl)	0-0.4	0.3	0.4				
Indirect Bilirubin (mg/dl)	0.1-0.8	0.5	0.4				
Alkaline Phosphatase (U/L)	64-306		248			284	
CRP			1+		3+		
Ferritin (ng/ml)	10-124						218
C-ANCA (U/ml)	<12 Negative						0.1
P-ANCA(U/ml)	<12 Negative						0.3
C3 (mg/dl)	90-180						116
C4 (mg/dl	10_40						21.7
Anti-ds DNA index	<0.9 Negative						0.5
A.Cardio (IgG)(U/ml)	<12 Negative						1.7
A.Cardio (IgM)(U/ml)	<12 Negative						6.9
Blood Culture			No Growth				
Urine culture				No Growth			
IgG4 (mg/l)	110-1570		1378.3				
Triglyceride (mg/dl)	Up to 200		67				

## Discussion

According to the American Gastroenterological Association Institute Guideline (AGA), the diagnosis of acute pancreatitis requires at least two of the following features: characteristic abdominal pain, and biochemical evidence of pancreatitis (amylase or lipase elevated >3 times the upper limit of normal); and/or radiographic evidence of pancreatitis on cross-sectional imaging (8). The described case in this case-report had acute pancreatitis. The infectious agents such as the different viruses, bacteria, parasites are known to induce this disorder (9). There are only a few studies that reported SARS CoV-2 a cause of acute pancreatitis, this case certainly leaves the possibility of Covid-19 as a cause of acute pancreatitis. Several factors can lead to an acute inflammatory process in the pancreas. Gallstone disease and alcohol abuse are responsible for most cases of acute pancreatitis. After exclusion of these causes, less frequent causes such as anatomic and functional abnormalities, metabolic conditions, drugs, trauma, infections, and vascular and genetic causes must be considered. In this respect for diagnosing etiology, we started investigation by obtaining detailed history of biliary disease or gallstone, former cholecystectomy, drug or alcohol abuse, personal history of dyslipidemia, recent abdominal surgery, trauma or endoscopic retrograde cholangiopancreatography (ERCP) and complete physical examination was done. According to recent guidelines, blood sample has been obtained to test liver function, calcium and triglyceride level. Abdominal ultrasound for probable biliary etiology was also done. As mentioned above, in this case, the etiologies of gallstones, alcohol and dyslipidemia ruled out, and in this regard, patients were classified as having an unclear etiology. An autoimmune etiology was also considered and serum IgG4 level was checked and it was within normal range. As the etiology still remained occult, biliopancreatic anatomic alterations and microlithiasis were also considered and in this regard, abdominal CT scan and magnetic resonance cholangiopancreatography (MRCP) were done and there were unremarkable findings. According to guideline of acute pancreatitis for diagnosing infectious etiology, a routine investigation is not advised unless high suspicions exist (9). The infectious agents’ such as different viruses, bacteria, parasites are known to induce this disorder (10). Due to the COVID-19 pandemic, we considered tests such as polymerase chain reaction (PCR) for COVID -19 and the test was positive. There are only a few studies that reported SARS CoV-2 a cause of acute pancreatitis, this case certainly leaves the possibility of Covid-19 as a cause of acute pancreatitis. This virus attached to cells with Angiotensin converting enzyme 2 (ACE2) receptor and via that, it can enter inside host cells. This enzyme binds to the membranes of various types of cells, so it can cause acute respiratory failure, gastrointestinal dysfunction, and liver dysfunction (4). Yuan Tian et al. reported the most common gastrointestinal presentation of COVID-19 disease as diarrhea and mentioned anorexia as the most frequent digestive symptoms in adults. Other gastrointestinal symptoms that present with this disease are abdominal pain, nausea and vomiting and even gastrointestinal bleeding (5). Mao et al. confirmed that patients may present gastrointestinal symptoms alone without respiratory features. The diagnosis of COVID-19 disease may be postponed in these patients (10). Rabice et al. also reported a case of pregnant woman who was first diagnosed with COVID-19 infection and then was presented with acute pancreatitis. They ruled out other causes of acute pancreatitis and then discussed about the possible role of COVID-19 virus in acute pancreatitis presentation (11). 

In conclusion, this case highlights acute pancreatitis as a suspected complication associated with COVID-19. We still do not know about the exact mechanism of inducing acute pancreatitis by COVID-19 virus. Further research is needed to explore the possibility of causation for acute pancreatitis with COVID-19.

## Clinical significance:

This case emphasizes the significance of assessing pancreas-specific plasma amylase in patients with COVID-19 and abdominal pain.

## References

[1] WHO (2020). World Health Organization.

[2] Guan W-J, Ni Z-Y, Hu Y (2020). Clinical characteristics of coronavirus disease 2019 in China. N Engl J Med.

[3] Wong SH, Lui RN, Sung JJ (2020). Covid-19 and the digestive system. J Gastroenterol Hepatol.

[4] Renu K, Prasanna PL, Valsala Gopalakrishnan A (2020). Coronaviruses pathogenesis, comorbidities and multi-organ damage- A review. Life Sci.

[5] Tian Y, Rong L, Nian W, He Y (2020). Review article: gastrointestinal features in COVID-19 and the possibility of faecal transmission. Aliment Pharmacol Ther.

[6] Forsmark CE, Vege SS, Wilcox CM (2016). Acute pancreatitis. New Engl J Med.

[7] Rawla P, Bandaru SS, Vellipuram AR (2017). Review of infectious etiology of acute pancreatitis. Gastroenterology Res.

[8] Crockett SD, Wani S, Gardner TB (2018). American gastroenterological association institute guideline on initial management of acute pancreatitis. Gastroenterology.

[9] Del Vecchio Blanco G, Gesuale C, Varanese M, Monteleone G, Paoluzi OA (2019). Idiopathic acute pancreatitis: a review on etiology and diagnostic work-up. Clin J Gastroenterol.

[10] Mao R, Qiu Y, He J-S (2020). Manifestations and prognosis of gastrointestinal and liver involvement in patients with COVID-19: a systematic review and meta-analysis. The Lancet Gastroenterol Hepatol.

[11] Rabice SR, Altshuler PC, Bovet C (2020). COVID-19 infection presenting as pancreatitis in a pregnant woman: A case report. Case Rep Women Health.

